# *Bacillus velezensis* FZB42 in 2018: The Gram-Positive Model Strain for Plant Growth Promotion and Biocontrol

**DOI:** 10.3389/fmicb.2018.02491

**Published:** 2018-10-16

**Authors:** Ben Fan, Cong Wang, Xiaofeng Song, Xiaolei Ding, Liming Wu, Huijun Wu, Xuewen Gao, Rainer Borriss

**Affiliations:** ^1^Co-Innovation Center for Sustainable Forestry in Southern China, College of Forestry, Nanjing Forestry University, Nanjing, China; ^2^Department of Biomedical Engineering, Nanjing University of Aeronautics and Astronautics, Nanjing, China; ^3^Department of Plant Pathology, College of Plant Protection, Nanjing Agricultural University, and Key Laboratory of Integrated Management of Crop Diseases and Pests, Ministry of Education, Nanjing, China; ^4^Institut für Biologie, Humboldt Universität Berlin, Berlin, Germany; ^5^Nord Reet UG, Greifswald, Germany

**Keywords:** *Bacillus velezensis*, FZB42, AmyloWiki, induced systemic resistance (ISR), non-ribosomal synthesized lipopeptides (NRPS), non-ribosomal synthesized polyketides (PKS), volatiles, plant growth promoting bacteria (PGPR)

## Abstract

*Bacillus velezensis* FZB42, the model strain for Gram-positive plant-growth-promoting and biocontrol rhizobacteria, has been isolated in 1998 and sequenced in 2007. In order to celebrate these anniversaries, we summarize here the recent knowledge about FZB42. In last 20 years, more than 140 articles devoted to FZB42 have been published. At first, research was mainly focused on antimicrobial compounds, apparently responsible for biocontrol effects against plant pathogens, recent research is increasingly directed to expression of genes involved in bacteria–plant interaction, regulatory small RNAs (sRNAs), and on modification of enzymes involved in synthesis of antimicrobial compounds by processes such as acetylation and malonylation. Till now, 13 gene clusters involved in non-ribosomal and ribosomal synthesis of secondary metabolites with putative antimicrobial action have been identified within the genome of FZB42. These gene clusters cover around 10% of the whole genome. Antimicrobial compounds suppress not only growth of plant pathogenic bacteria and fungi, but could also stimulate induced systemic resistance (ISR) in plants. It has been found that besides secondary metabolites also volatile organic compounds are involved in the biocontrol effect exerted by FZB42 under biotic (plant pathogens) and abiotic stress conditions. In order to facilitate easy access to the genomic data, we have established an integrating data bank ‘AmyloWiki’ containing accumulated information about the genes present in FZB42, available mutant strains, and other aspects of FZB42 research, which is structured similar as the famous SubtiWiki data bank.

## Introduction and Short History of Gram-Positive PGPR Research

Bacteria that are associated with plant roots and exert beneficial effects on plant development are referred to as plant-growth-promoting rhizobacteria (PGPR; Kloepper et al., 1980). It is well accepted today, that numerous PGPR are also enabled to control plant diseases.

Main subject of present and past research about microbial inoculants with beneficial action on plant health and growth are plant-associated representatives of the bacterial genus *Pseudomonas*, known as strong and persistent colonizer of plant roots (Burr et al., 1978). However, its commercial use is limited by difficulties in preparing stable and long-living bioformulations. As early as at the end of the 19th century a bacterial soil-fertilizing preparation Alinit^®^ consisting of spores of the soil bacterium *Bacillus ellenbachensis*, later reclassified as *Bacillus subtilis*, was introduced by the German landowner Albert Caron (1853–1933) on his estate in Ellenbach (Caron, 1897). Alinit was marketed as “bacteriological fertilizer for the inoculation of cereals” by “*Farbenfabriken* former Friedrich Bayer,” the later Bayer AG, in Elberfeld, Germany. The history of these early attempts in using bacterial inoculants is comprehensively described by Kolbe (1993). After a long period of silence, the plant-growth-promoting effect of *Bacillus* spp. was rediscovered in Broadbent et al. (1977). Today, formulations based on plant-beneficial endospore-forming Bacilli are by far the most widely used agents on the biopesticide market (Borriss, 2011). Especially, members of the *B. subtilis* species complex (rRNA group 1) which includes at present more than 20 closely related species (Fan et al., 2017a), and, to a minor extent, of the genus *Paenibacillus* spp., are able to suppress efficiently plant pathogens, such as viruses, bacteria, fungi and nematodes in vicinity of plant roots. This review describes the current ‘state of the art’ of the model strain for PGPR – and biocontrol, *Bacillus velezensis* FZB42, and the integrative data bank ‘AmyloWiki,’ recently established for this bacterium.

FZB42 (=BGSC 10A6, DSM23117), the prototype of gram-positive bacteria with phytostimulatory and biocontrol action, has been genome sequenced in Chen et al. (2007) and is subject of intensive research. Since its isolation from beet rhizosphere (Krebs et al., 1998) more than 140 articles about FZB42 have been published^1^. FZB42 and its closely related ‘cousin’ FZB24, are successfully used as biofertilizer and biocontrol bacteria in agriculture being especially efficient against fungal and bacterial pathogens^2^. Beneficial effects of FZB42/FZB24 on plant growth and disease suppression in field trials were reported for potato (Schmiedeknecht et al., 1998), cotton (Yao et al., 2006), strawberry (Sylla et al., 2013), wheat (Talboys et al., 2014), lettuce (Chowdhury et al., 2013), and tomato (Elanchezhiyan et al., 2018), for example.

**FIGURE 1:**
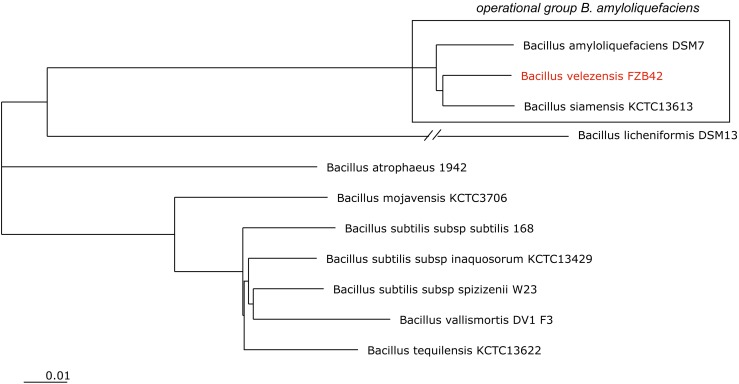
NJ phylogenomic tree, constructed from 11 type strain genomes with highest similarity to B. subtilis 168^T^. The genome of B. licheniformis DSM13 was used as outgroup. The tree was build out of a core of 1946 genes per genome, 21406 in total. The core has 586283 AA-residues/bp per genome, 6449113 in total. B. velezensis FZB42 (labeled in red) is a member of the operational group B. amyloliquefaciens (boxed). The scale bar corresponds to 0.01 substitutions per site.

In past, FZB42 and related phytostimulatory Bacilli were subjects of intensive efforts to clarify their taxonomic position. The group of plant-associated, endo-spore forming rhizobacteria (Reva et al., 2004) is known as member of the *B. subtilis* species complex (Fritze, 2004), which included originally *B. subtilis*, *B. licheniformis*, and *B. pumilus* (Gordon et al., 1973). In 1987, the species *B. amyloliquefaciens* (Priest et al., 1987) was added, and FZB42 and some other biocontrol bacteria were found as belong to this species (Idriss et al., 2002). By taking advantage of availability of an increasing number of genome sequences, we distinguished two subspecies: *B. amyloliquefaciens* subsp. *amyloliquefaciens* (type strain DSM7^T^) and *B. amyloliquefaciens* subsp. *plantarum* (type strain FZB42^T^) (Borriss et al., 2011). According to extended phylogenomic analysis *B. amyloliquefaciens* subsp. *plantarum* was shown as a later heterotypic synonym of *B. velezensis* (Dunlap et al., 2016), Recently, we proposed to establish an “operational group *B. amyloliquefaciens*,” which includes *B. amyloliquefaciens*, known for its ability to produce industrial enzymes (amylases, glucanases and proteases), *B. siamensis*, mainly occurring in Asian food, and PGPR *B. velezensis*, the main source for bioformulations increasingly used in agriculture for protecting plant health and to stimulate plant growth (Fan et al., 2017a, **Figure 1**).

## FZB42, the Gram-Positive Prototype for Biocontrol of Plant Pathogens

Biocontrol effects exerted by *B. velezensis* FZB42 and other antagonistic acting Bacilli are due to different mechanisms: besides direct antibiosis and competition by secretion of a spectrum of secondary metabolites in the rhizosphere (Borriss, 2011), the beneficial action on the host-plant microbiome (Erlacher et al., 2014), and stimulation of plant induced systemic resistance (ISR, Kloepper et al., 2004; Chowdhury et al., 2015a) are of similar importance.

Remarkably, in contrast to Gram-negative biocontrol bacteria and fungal plant pathogens, application of FZB42 did not lead to durable changes in composition of rhizosphere microbial community (Chowdhury et al., 2013; Kröber et al., 2014). Moreover, application of FZB42 was shown to compensate negative changes within composition of the root microbiome caused by plant pathogens (Erlacher et al., 2014).

Induced systemic resistance is triggered by a range of secondary metabolites, which are called ‘elicitors.’ Different signaling pathways, such as jasmonic acid (JA), ethylene (ET), and salicylic acid (SA) are activated to induce plant resistance. Mutant strains of FZB42, devoid in synthesis of surfactin (srf), were found impaired in triggering of JA/ET dependent ISR in lettuce plants, when challenged with plant pathogen *Rhizoctonia solani* (Chowdhury et al., 2015b). The lower expression of the JA/ET-inducible plant defensin factor (PDF1.2) in a *sfp* mutant strain, completely devoid in non-ribosomal synthesis of lipopeptides and polyketides, compared to the *srf* mutant strain, only impaired in surfactin synthesis, suggests that secondary metabolites other than surfactin might also trigger plant response.

Gray leaf spot disease caused by *Magnaporthe oryzae* is a serious disease in perennial ryegrass (*Lolium perenne*). A mutant strain of FZB42 (AK3) only able to produce surfactin but no other lipopeptides such as bacillomycin D, and fengycin was shown to induce systemic resistance (ISR). Similarly, treatment with crude surfactin suppressed the disease in perennial ryegrass. ISR defense response was found connected with enhanced hydrogen peroxide (H_2_O_2_) development, elevated cell wall/apoplastic peroxidase activity, and deposition of callose and phenolic/polyphenolic compounds. Moreover, a hypersensitive response reaction and enhanced expression of different defense factors, such as peroxidase, oxalate oxidase, phenylalanine ammonia lyase, lipoxygenase, and defensins were caused by surfactin and also the surfactin producing mutant strain (Rahman et al., 2015).

Recent studies performed with mutant strains of *B. velezensis* SQR9, which is closely related with FZB42, revealed that non-ribosomal synthesized lipopeptides fengycin and bacillomycinD, the non-ribosomal synthesized polyketides macrolactin, difficidin, and bacillaene, the dipeptide bacilysin, exopolysaccharides, and volatile organic compounds (VOCs) contribute to ISR response in *Arabidopsis* plantlets after infection with plant pathogens *Pseudomonas syringae* pv. tomato and *Botrytis cinerea* (Wu G. et al., 2018).

Volatile organic compounds produced by *B. velezensis* GB03 have been reported to trigger synthesis of ET/JA-responsive plant defense gene PDF1.2 (Ryu et al., 2004; Sharifi and Ryu, 2016). Thirteen VOCs produced by FZB42 were identified using gas chromatography-mass spectrometry analysis. A direct effect against plant pathogens was registered: benzaldehyde, 1,2-benzisothiazol-3(2 H)-one and 1,3-butadiene significantly inhibited the colony size, cell viability, and motility of *Ralstonia solanacearum*, the causative agent of bacterial wilt in a wide variety of potential host plants (Tahir et al., 2017). Furthermore, transcription of type III (T3SS) and type IV secretion (T4SS) systems were down regulated. In addition, synthesis of other genes contributing to pathogenicity, such as *eps*-genes responsible for extracellular polysaccharides, and genes involved in chemotaxis (*motA, fliT*) were found repressed. Simultaneously, the VOCs significantly up-regulated the expression of plant genes related to wilt resistance and pathogen defense. Over-expression of plant defense genes *EDS1* and *NPR1* suggested that the SA pathway is involved in the ISR response elicited by surfactin (Tahir et al., 2017).

A recent analysis performed with FZB42 VOCs confirmed that signal pathways involved in plant systemic resistance were positively affected. JA response (VSP1 and PDF1.2) and SA response genes (PR1 and FMO1) were triggered in *Arabidopsis* plantlets after incubation with the volatiles. Noteworthy, defense against nematodes were elicited by volatiles in *Arabidopsis* roots (Hao et al., 2016).

An interesting mechanism of FZB42 to avoid leaf pathogen infection has been recently described. The foliar pathogen *Phytophthora nicotianae* is able to penetrate inside of plant tissues by using natural entry sites, such as stomata. Recently it was shown that colonizing of plant roots by FZB42 restricted entry of the pathogen into leave tissues of *Nicotiana benthamiana.* It was found that FZB52 turned on the abscisic acid (ABA) and SA-regulated pathways to induce stomatal closure after pathogen infection. In addition, it was shown, that several SA- and JA/ET-responsive genes in the leaves became activated in presence of FZB42, suggesting that these signaling pathways are also contributing to plant defenses against *P. nicotianae* (Wu L. et al., 2018).

Besides their indirect action against pathogens via triggering of ISR, polyketides and lipopeptides act directly against bacterial and fungal plant pathogens. They comprise two families of secondary metabolites non-ribosomally synthesized by multimodular enzymes, polyketide synthases (PKSs) and Peptide synthetases (NRPS), acting in assembly line arrays. The monomeric building blocks are either organic acids (polyketides) or amino acids (lipopeptides), respectively (Walsh, 2004). Their synthesis is depending on an enzyme (Sfp) that transfers 4′-phosphopantheine from coenzyme A to the carrier proteins of nascent peptide or polyketide chains. In Bacilli, e.g., FZB42, a special class of PKSs that lacks the cognate AT domain and require a discrete AT enzyme acting iteratively *in trans* (trans AT) was detected (Shen, 2003). The broadly conserved antiterminator protein LoaP (Nus G family) was identified as regulator of macrolactin and difficidin gene clusters *in B. velezensis* FZB42 on the level of transcription elongation (Goodson et al., 2017). Unfortunately, structural instability of these polyketides excluded their use as antibacterial agents.

Lipopeptides are another important class of secondary metabolites, also non-ribosomally synthesized by giant multifunctional enzymes (peptide synthetases, NRPS). Similar to PKS, three catalytic domains are involved in each elongation cycle: (1) The A-domain (adenylation domain) select its cognate amino acid; (2) The PCP domain (peptidyl-carrier domain) is equipped with a PPan prosthetic group to which the adenylated amino acid substrate is transferred and bound as thioester; (3) The condensation domain (C-domain) catalyzes formation of a new peptide bond (Duitman et al., 1999). The lipopeptide bacillomycin D is an efficient antifungal compound produced by FZB42. Its 50% effective concentration against the fungal pathogen *Fusarium graminearum* was determined to be approximately 30 μg/ml. Bacillomycin D induced morphological changes in the plasma membranes and cell walls of *F. graminearum* hyphae and conidia. Furthermore, bacillomycin D induced the accumulation of reactive oxygen species and caused cell death in *F. graminearum* hyphae and conidia. Bacillomycin D suppresses *F. graminearum* on corn silks, wheat seedlings, and wheat heads (Gu et al., 2017).

## The Genomes of FZB42 and *B. subtilis* 168, a Comparison

**FIGURE 2:**
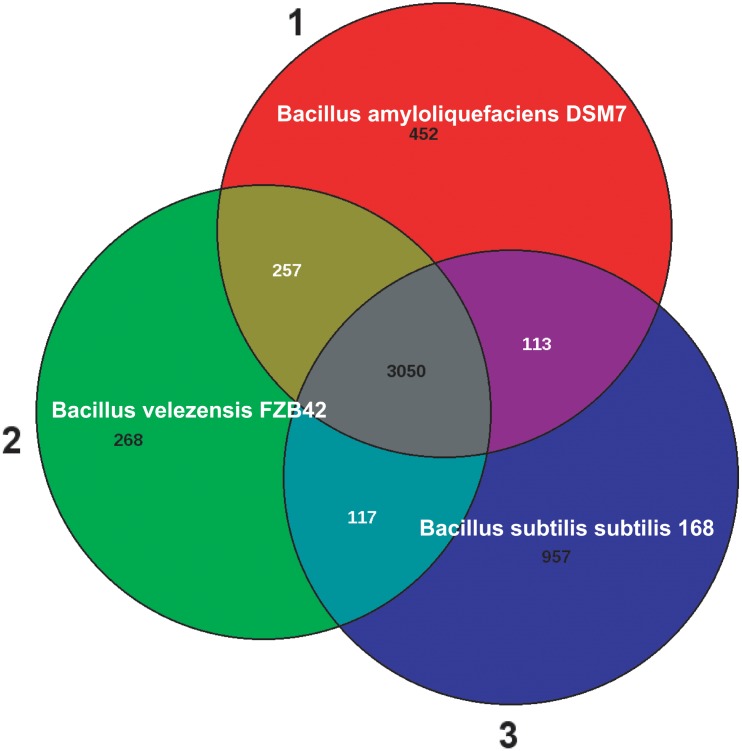
The Venn diagram of *Bacillus amyloliquefaciens* DSM7 **(1)**, *Bacillus velezensis* FZB42 **(2)**, and *Bacillus subtilis subtilis* 168 **(3)**. The numbers of reciprocal best hits between subsets of genomes are shown. Note, 100% identical paralogous genes were not counted in the Venn diagram numbers (Blom et al., 2016). The three strains share 3050 genes according to the best hit calculation, whilst 268 genes were found unique in FZB42. A direct comparison between FZB42 and *B. subtilis* 168 revealed that they have 3122 genes in common, whilst 522 genes were found unique in FZB42.

Today, *B. subtilis* is considered as being a plant-associated bacterium (Wipat and Harwood, 1999; Borriss et al., 2018). A direct comparison between the genomes of *B. subtilis* 168 and *B. velezensis* FZB42 (**Table 1**) revealed that 534 FZB42 genes are not occurring in *B. subtilis* 168, but 3158 genes are shared between both species. By contrast, there are only 423 singletons defined for FZB42 *vs. Bacillus subtilis* 168. In this context one has to mention, that the singleton numbers don’t correspond to the numbers in the Venn diagram. The Venn diagram (**Figure 2**) shows the numbers of reciprocal best hits between subsets of genomes. However, a gene without reciprocal best hit to another genome does not necessarily have to be a singleton. A singleton is defined as a gene without any hit against any other genome than the own one.

**Table 1:** Comparison of the genomes of *Bacillus subtilis 168* (domesticated), *Bacillus subtilis 3610* (wild type), *Bacillus amyloliquefaciens* DSM7 (non-plant associated), and *Bacillus velezensis* FZB42 (plant associated).

	*B. subtilis* 168	*B. subtilis* 3610	*B. amyloliquefaciens* DSM7	*B. velezensis* FZB42
NCBI accession	AL009126.3	*NZ_CM000488.1*	NC_014551.1	NC_009725
Size (bp)	4,215,606	4,214,598	3,980,199	3,918,589
ANIb (AL009126.3)	^∗^	100.00 (99.61)	76.43 (72.32)	76.35 (75.15)
ANIb (NC_009725)	76.49 (69.21)	76.47 (69.24)	93.84 (85.28)	^∗^
Transcription units	1609	2837	2584	2506
Total genes	4381	4398	3999	3808
Protein genes	4194	4283	3870	3687
RNA genes	187	115	128	120
tRNAs	86	91	94	88
Pseudogenes	70	0	121	84
Pathways	246	184	245	238
Enzymatic reactions	1217	768	1489	1446
Transport reactions	51	80	157	150
Polypeptides	4235	4284	3870	3687
Protein features	3890	3610	3389	3305
Protein complexes	188	42	51	20
Enzymes	962	697	1114	1041
Transporters	51	134	162	175
Compounds	992	679	1257	1267
**Non-ribosomal synthesized secondary metabolites**				
BGC0000433	Surfactin^1^ 356968 – 422359	Surfactin 356500 – 421899	Surfactin 313124 – 378534	Surfactin 322618 – 388025
BGC0000181	–	–	–	Macrolactin 1374169 – 1460068
BGC0001089	Bacillaene^1^ 1768695 – 1878521	Bacillaene 1767850 – 1877685	Bacillaene 1773732 – 1876436	Bacillaene 1688756 – 1791439
BGC0001103/ BGC0001090	–	–	Iturin/Mycosubtilin 1968514 – 2008850	Bacillomycin D 1851172 – 1988997
BGC0001095	Fengycin^1^ 1934525 – 2017957	Fengycin 1933702 – 2017134	Fengycin^2^ 1948515 – 2058936	Fengycin 1851172 – 1988997
Triketide pyrone	T3pks 2189857 – 2191463	T3pks 2296123 – 2337238	T3pks 2170363 – 2211463	T3pks 2122078–2123684
BGC0000176	–	–	–	Difficidin 2260090 – 2360537
Nrps2	–	–	Orphan Nrps2 2500781 – 2552402	–
Nrps1	–	–	–	Orphan Nrps1 2885927 – 2868410
BGC0000309	Bacillibactin^1^ 3260519 – 3310260	Bacillibactin 3259511 – 3309252	Bacillibactin 3033649 – 3100417	Bacillibactin 3001250 – 3068038
BGC0001184	Bacilysin 3850668 – 3892086	Bacilysin 3849661 – 3891079	Bacilysin 3636549 – 3677967	Bacilysin 3576267 – 3617685
**Ribosomal synthesized antimicrobial compounds (RiPPs)**				
BGC0000558	Sublancin 2259521 – 2279691	Sublancin 2258687 – 2278857	–	–
BGC0000602	Subtilosin_A 3826058 – 3847669	Subtilosin_A 3825052 – 3846663	–	–
BGC0000616	–	–	Amylocyclicin 3076887 – 3081038	Amylocyclicin 3044505 – 3048679
BGC0000569	–	–	–	Plantazolicin 726469 – 736360
Antibacterial peptide Lci	–	–	Lci 1296288- 1296563	Lci 310858 - 311142

*Phylogenomic relationship was determined by ANIb (average nucleotide identity, JSpeciesWS, Richter et al., 2016), either using B. subtilis 168 or B. velezensis for comparison. General data were taken from MetaCyc data base (Caspi et al., 2018). Gene clusters encoding secondary metabolites were identified by antiSMASH version 4.1.0 (Blin et al., 2017). The MIBiG accession numbers (Medema et al., 2015) are indicated. ^1^Not expressed in B. subtilis 168, but expressed in its wild type counterpart B. subtilis 3610. ^2^Fengycin gene cluster is only fragmentary in DSM7. Not expressed in DSM7 (Borriss et al., 2011). ^∗^Means that ANIb analyses performed with B. subtilis 168 (AL009126.3) and FZB42 (NC_009725) against itself results in 100% identity.*

Many genes, essential for a plant-associated lifestyle, are shared between *B. subtilis* 168 and FZB42 as well. Relevant examples are YfmS, a chemotaxis sensory transducer, which is involved in plant root colonization (Allard-Massicotte et al., 2017), and BlrA (formerly YtvA) a blue light receptor related to plant phototropins (Borriss et al., 2018). However, due to a century of ‘domestication’ under laboratory conditions, the type strain *B. subtilis* 168 has lost its ability to colonize roots and to control plant diseases. Its ability to form biofilms on solid surfaces (e.g., rhizoplane) is attenuated by several mutations detected in the genes *sfp* (necessary for production of lipopeptides and polyketides), *epsC* (required for extracellular polysaccharide synthesis), *swrA* (essential for swarming differentiation on solid surfaces), and *degQ*, which stimulates phosphorylation of DegU. By contrast, the closely related wild type *B. subtilis* 3610 forms robust biofilms and is able to produce antimicrobial compounds (**Table 1**). It was shown that by introducing wild type alleles of these four genes and the spo0F phosphatase encoding *rapP* gene, residing on a large plasmid occurring in *B. subtilis* 3610 but not in *B. subtilis* 168, the laboratory strain 168 forms biofilms which are essentially the same as in 3610. This demonstrates that domestication of *B. subtilis* 168 is only due to the four gene mutations mentioned above and loss of the plasmid occurring in strain 3610 (McLoon et al., 2011). Notably, FZB42 does not harbor a *rapP* containing plasmid, but is able to produce robust biofilm similar to *B. subtilis* 3610.

FZB42 releases several cellulases and hemicellulases degrading the external cellulosic and hemicellulosic substrates present in plant cell walls. Final products of enzymatic hydrolysis are free oligosaccharides, which act as elicitors of plant defense (Ebel and Scheel, 1997). Some genes encoding extracellular hydrolases, such as *amyE* (alpha-amylase), *eglS* (endo-1,4-β-glucanase), and *xynA* (xylanase) occurred only in the plant-associated representatives of the ‘*B. amyloliquefaciens* operational group’ but not in their soil-associated counterparts (Borriss et al., 2011; Zhang et al., 2016). Similarly, an operon involved in xylan degradation (*xylA, xynP, xynB, xylR)* is present in *B. subtilis* 168 and *B. velezensis* FZB42 but not in *B. amyloliquefaciens* DSM7^T^ suggesting that both strains have in common some genes involved in plant macromolecule degradation (Rückert et al., 2011).

*Bacillus velezensis* harbored additional genes involved in hexuronate (galacturonate and fructuronate) degradation. Three genes were found unique for *B. velezensis* FZB42 and other members of this species: *kdgK1*, (2-dehydro-3-deoxygluconokinase), *kdgA* (2-dehydro-3-deoxyphosphogluconate aldolase), and the transcription regulator *kdgR.* They are part of the six-gene *kdgKAR* operon (He et al., 2012). In addition *yjmD*, a gene with putative galacticol-1-phosphate dehydrogenase function and two further genes: *uxuA* encoding mannonate dehydratase, and *uxuB* encoding mannonate oxidoreductase are part of the six-gene transcription unit. A second operon, containing the genes *uxaC*, *uxaB*, and *uxaA* encoding enzymes for metabolizing different hexuronates to D-altronate and D-fructuronate, occurs distantly from the *kdgAR* operon. In *Escherichia coli* K12 UxuA, KdgK, and KdgA are involved in a degradative pathway of aldohexuronates (Portalier et al., 1980). Whilst the complete biochemical pathway from galacturonate to KDG is present, no gene encoding D-glucuronate isomerase was detected, suggesting that *B. velezensis* is not able to metabolize D-glucuronate (He et al., 2012).

Nearly 10% of the FZB42 genome is involved in synthesizing antimicrobial compounds, such as the polyketides bacillaene, macrolactin and difficidin (Chen et al., 2006; Schneider et al., 2007) and the lipopeptides surfactin, bacillomycin D and fengycin (Koumoutsi et al., 2004). In total, the FZB42 genome harbors 13 gene clusters devoted to non-ribosomal and ribosomal synthesis of secondary metabolites with putative antimicrobial action. In two cases, in the *nrs* gene cluster and in the type III polyketide gene cluster their products are not identified till now (**Table 1**). Similar to *B. subtilis* 168^T^, the genome of the non-plant associated soil bacterium *B. amyloliquefaciens* DSM7^T^ possesses a much lower number of gene clusters involved in synthesis of antimicrobial compounds than FZB42 (**Table 1**).

Notably, the gene clusters involved in non-ribosomal synthesis of the antifungal lipopeptides bacillomycin D and fengycin, and the polyketides difficidin and macrolactin are missing or fragmentary in DSM7^T^ and other representatives of *B. amyloliquefaciens* suggesting that synthesis of these secondary metabolites might be important for the plant associated life style. Five out of a total of 13 gene clusters are located within variable regions of the FZB42 chromosome, suggesting that they might be acquired *via* horizontal gene transfer (Rückert et al., 2011). Most of them (bacillomycin D, macrolactin, difficidin, plantazolicin, and the orphan *nrsA-F* gene cluster) are without counterpart in DSM7^T^ and *B. subtilis* 168^T^. Moreover, it has been shown experimentally that DSM7^T^, due to a deletion in the fengycin gene cluster, is unable to produce fengycin (Borriss et al., 2011), notably the gene cluster for synthesis of iturinA is present in the DSM7^T^ genome (**Table 1**).

Besides type I PKS also genes encoding type III polyketide synthases are present in the genome of FZB42. By contrast to type I PKSs, type III PKSs catalyze priming, extension, and cyclization reactions iteratively to form a huge array of different polyketide products (Yu et al., 2012). In *B. subtilis* gene products of *bspA-bspB* operon were functionally characterized, and found to be involved in synthesis of triketide pyrones. The type III PKS BspA catalyzes synthesis of triketide pyrones and BspB (YpbQ) is a methyltransferase catalyzing its posttranslational modification to alkylpyrones ethers (Nakano et al., 2009). However, their biological role needs further elucidation. Orthologs of *bspA* and *bspB* are present in FZB42 and DSM7^T^ (**Table 1**).

Another group of secondary metabolites are bacteriocins, which represent a class of post-translationally modified peptide antibiotics (Schnell et al., 1988). Together with peptides without antibiotic activity, they are generally termed RiPPs (ribosomally synthesized and post-translationally modified peptides). RiPP precursor peptides are usually bipartite, being composed of an *N*-terminal leader and *C*-terminal core regions. RiPP precursor peptides can undergo extensive posttranslational modification, yielding structurally and functionally diverse products (Burkhart et al., 2015). In recent years, two RiPPs with antibacterial activity (bacteriocins) were identified in FZB42: plantazolicin (Scholz et al., 2011) and amylocyclicin (Scholz et al., 2014).

An antibacterial substance still produced by a FZB42 mutant strain, unable to synthesize non-ribosomally any antimicrobial compound, was identified together with the gene cluster responsible for its biosynthesis. The *pzn* genes cluster encodes a small precursor peptide PznA that is post-translationally modified to contain thiazole and oxazole heterocycles. These rings are derived from Cys and Ser/Thr residues through the action of a modifying “BCD” synthetase complex, which consists of a cyclodehydratase (C), a dehydrogenase (B), and a docking protein (D) (Scholz et al., 2011). After modification and processing of the precursor peptide plantazolicin contains an unusual number of thiazoles and oxazoles (Kalyon et al., 2011). The structure variant plantazolicin A inhibits selectively *Bacillus anthracis* (Molohon et al., 2016), and is efficient against plant pathogenic nematodes (Liu et al., 2013), whilst the precursor molecule PZNB is inactive (Kalyon et al., 2011).

The head-to-tail cyclized bacteriocin amylocyclicin was firstly described in *B. amyloliquefaciens* FZB42 (Scholz et al., 2014). Circular bacteriocins are non-lanthionine containing bacteriocins with antimicrobial activity against Gram-positive food-borne pathogens (van Belkum et al., 2011). Amylocyclicin was highly efficient against Bacilli, especially against a *sigW* mutant of *B. subtilis* (Y2) (Butcher and Helmann, 2006). An orthologous gene cluster was also detected in *B. amyloliquefaciens* DSM7^T^ (**Table 1**).

Lci was reported as an antimicrobial peptide synthesized by a *B. subtilis* strain with strong antimicrobial activity against plant pathogens, e.g., *Xanthomonas campestris* pv. *oryzae* and *Pseudomonas solanacearum* PE1. Its solution structure has a novel topology, containing a four-strand antiparallel β-sheet as the dominant secondary structure (Gong et al., 2011). The gene is not present in the *B. subtilis* 168 genome, but was detected in FZB42 and *B. amyloliquefaciens* DSM7^T^ (**Table 1**). Lci was found highly expressed in FZB42 biofilms (Kröber et al., 2016).

## FZB42 Gene Expression is Affected by Plants and Vice Versa

Nowadays, global gene expression studies were increasingly performed to enlarge our knowledge base about effect of plants on gene expression in Gram-positive plant associated bacteria (Borriss, 2015a). The first combined transcriptome- and proteome analysis in *Bacillus*, using both, DNA-microarrays and 2-D protein gel electrophoresis, was conducted with *B. subtilis* 168 (Yoshida et al., 2001). Plant-bacteria interactions were studied with *B. subtilis* OKB105 in presence of rice seedlings. Transcriptome analysis revealed that expression of 176 bacterial genes was affected by the host plant (Shanshan et al., 2015).

In this context several studies were performed with FZB42, too. Transcription of many genes involved in carbon and amino acid metabolism was turned on, when maize root exudates were added to FZB42 cells growing in planktonic culture suggesting that nutrients present in root exudates are utilized by bacteria cells (Fan et al., 2012). Dependency of FZB42 from nutrient sources present in root exudates was corroborated in a second transcriptome study performed with DNA-microarrays. In this case root exudates with different composition obtained from maize plantlets growing under stress conditions (N, P, Fe, and K limitation) were used. In case of root exudates obtained from N-deprived maize plantlets containing decreased amounts of aspartate, valine and glutamate, FZB42 cells were found to be downregulated in transcription of genes involved in protein synthesis indicating a general stress response. By contrast, P-limited root exudates led to enhanced transcription of FZB42 genes involved in motility and chemotaxis, possibly suggesting a chemotactic response toward carbohydrates in root exudates (Carvalhais et al., 2013). Transcriptional profiling via RNA-sequencing in the taxonomically related *B. velezensis* SQR9 revealed that maize root exudates stimulated at first expression of metabolism-relevant genes and then genes involved in production of the extracellular matrix (Zhang et al., 2015).

Response of FZB42 on maize root exudates during late exponential and stationary growth phase was also investigated on the level of protein synthesis applying 2-D gel electrophoresis and MALDI TOF MS for protein identification. Elicitors of plant innate immunity such as flagellins, elongation factor Tu, and cold shock proteins were detected in the extracellular fluid (Kierul et al., 2015). Corresponding to the results obtained in our transcriptome studies, we found that the expression of genes involved in utilization of nutrients and transport was enhanced in presence of root exudates. The protein with the highest secretion in presence of maize root exudates was acetolactate synthase AlsS, an enzyme involved in post-exponential phase synthesis of acetoin and 2,3 butandiol (Kierul et al., 2015).

On the other hand, plants are also affected in their gene expression, when colonized by bacteria including representatives of *the B. amyloliquefaciens* operational group. Transcript analysis of rape seedlings confronted with a root-colonizing *B. velezensis* strain revealed that gene expression was more affected in leaves than in roots. Altogether the treatment caused a metabolic reprogramming in plant leaves (Bejai et al., 2009; Sarosh et al., 2009). Similar effects on plant gene expression were reported for root-colonizing *B. subtilis* FB17. A microarray study performed with *Arabidopsis* plantlets exposed to FB17 showed that expression of auxin-regulated genes and genes involved in metabolism, stress response and plant defense were up-regulated. Some *Arabidopsis* mutants deficient in three of the up-regulated genes, were found less colonized by FB17 (Lakshmanan et al., 2013). Further papers reporting about triggering of ISR response in plants by lipopeptides and VOCs from *B. velezensis* (Chowdhury et al., 2015b; Wu G. et al., 2018) were already discussed in a previous section.

Another study performed with FZB42 revealed that gene expression is dependent on life style. Ability to form biofilms is essential for colonizing plant root surfaces. Differential gene expression suggested that under biofilm-forming conditions transcription of 331 genes was increased and of 230 genes was decreased (Kröber et al., 2016).

The differential RNA-sequencing (dRNA-seq) technology was employed to unveil the structure of the FZB42 transcriptome (Fan et al., 2015). The unique feature of this technique is that two libraries split from the same RNA sample are compared. One library is subjected to terminator exonuclease that preferentially degrades processed RNAs with 5′-monophosphate group, thus primary transcripts with 5′-triphosphate group are enriched in relative terms (Sharma et al., 2010). Applying this method, we obtained the first global transcription start sites (TSSs) map of a PGPR *Bacillus* species. We determined a comprehensive transcriptome profile for FZB42 by identifying 4,877 TSSs for protein-coding genes. This includes >2,000 primary TSSs, >700 secondary TSSs, and nearly 200 orphan TSSs. The primary TSSs have been identified for 60% of all FZB42 genes. In addition, >1,300 internal TSSs and >1,400 antisense TSSs were also identified. A lot of coding genes were shown to be transcribed from multiple TSSs and perhaps own different UTRs. Some mRNAs contained overlapped transcripts (Fan et al., 2015). The global charting of FZB42 TSSs can favor the identification of promoter regions, *cis*-acting regulatory elements, and cognate transcriptional regulators.

By applying the dRNA-seq technique differentially expressed genes under different growth conditions were identified. For example, a large group of genes that are specifically regulated by root exudates during stationary growth were identified. The results obtained extended and corroborated our previous results obtained by using microarrays (Fan et al., 2012). Knowledge of the genes affected in their expression by plant root exudates contributes to our understanding of rhizobacterial physiology and its interaction with their host plants. They are listed as ‘Interaction with plants’ in AmyloWiki^3^. Moreover, this study allowed us to propose 46 previously unrecognized genes. 78 polycistronic transcripts covering 210 genes were identified and 10 previously mis-annotated genes were corrected (Fan et al., 2015).

## Non-Coding Small RNAs

Over the last decade, a growing number of non-coding regulatory small RNAs (sRNAs) have been identified in bacteria (Li et al., 2013), although the functions of most of them are still unknown. Most of sRNAs do not encode a protein, but function as an RNA regulator directly targeting multiple mRNAs. It is revealed that many sRNAs contribute to bacterial adaptation to changing environments and growth conditions (Thomason et al., 2012), therefore it is feasible to expect that sRNAs may also coordinate mutual effects of rhizobacteria on plants.

Besides graphing the profile of expressed protein-coding genes, dRNA-seq technology also offers a possibility to identify genome-wide sRNAs. We detected hundreds of non-coding RNAs in FZB42, including 136 antisense RNAs, 53 *cis*-encoded leader sequence or riboswitches, and 86 sRNA candidates (Fan et al., 2015). Among them 21 sRNAs were further validated by Northern blotting. According to their gene positions, the majority of the sRNAs perhaps act in-trans targeting the mRNAs encoded from a distant locus. Generally, sRNAs often binds to their target mRNAs, at 5′UTR in many cases, and thus modulate mRNA translation (Waters and Storz, 2009). Since the genome-wide TSS annotation of FZB42 informs about potential sRNA target sites of mRNAs, our study has provided a valuable basis for studying rhizobacterial sRNA regulation.

The function of the identified sRNAs has not been characterized in detail. However, some of the sRNAs were found related to a specific growth phase or to respond to environmental cues (soil extract or maize root exudates) (Fan et al., 2015). Furthermore, one sRNA was found to be involved in *Bacillus* sporulation and biofilm formation (data not shown). Since, sRNAs are more studied in Gram-negative than in Gram-positive bacteria, systematic detection of sRNAs in FZB42 extends our knowledge base about plant-associated Gram-positive bacteria, especially to rhizobacteria–plant interactions.

## Protein Modification

In recent years post-translational modifications (PTM) of proteins, such as protein phosphorylation, acetylation, methylation, and succinylation, attracted increasing attention due to their important physiological significance in organisms (Choudhary et al., 2014). Whereas most studies of PTM were performed in eukaryotic cells, nowadays the role of PTM in prokaryotes is increasingly investigated. Acetylation of lysine residues in FZB42 was studied using a combination of immune-affinity purification and high- resolution LC-MS/MS. A total of 3,268 acetylated lysine residues were detected in 1254 proteins, accounting for 32.9% of the entire proteins of FZB42. Remarkably, a high proportion (71.1 and 78.6%) of the proteins related to the synthesis of polyketides and lipopeptides were found acetylated. The finding implies an important role of lysine acetylation in the regulation of FZB42 antibiotic biosynthesis (Liu et al., 2016).

**FIGURE 3:**
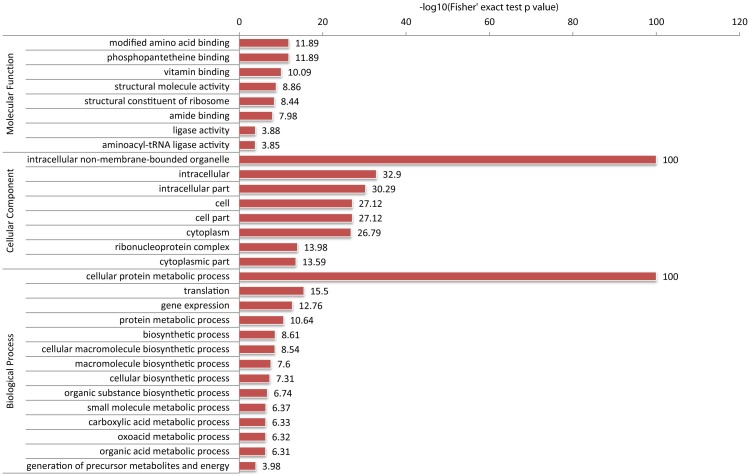
Distribution of FZB42 malonylated proteins in various functional categories according to the GO database. The ratio of Kmal sites located in the protein to all KmaI sites was compared with the ratio of malonylated proteins to all proteins in the database. The one-tailed Fisher’s exact test was used to test the enrichment and the result with *p*-value < 0.05 is considered significant.

Using a similar technique, we profiled lysine malonylation of proteins in FZB42. In total, we identified 809 malonyl-lysine sites in 382 proteins (**Figure 3**). Lysine malonylation targets the proteins implicated in a wide range of biological functions, such as fatty acid biosynthesis and metabolism, central carbon metabolism, translation processes, and NAD(P) binding. A group of proteins involved in bacterium-plant interaction was also malonylated. Moreover, malonylation seems to occur on proteins with higher surface accessibility, although the significance of the site preference remains unclear. Similar to lysine acetylation, 33 polyketide synthases (PKS) and polypeptide synthetases (NRPS) involved in non-ribosomal synthesis of bacillaene, difficidin, macrolactin, and bacillomycinD, fengycin and surfactin, were found highly malonylated. They account for 8.6% of all malonylated proteins. The PKSs and NRPSs possessed 128 malonylation sites, averagely 3.8 sites per protein, which is significantly higher than the mean of 2.1 malonylation sites per protein. The polyketide synthases, BmyA, BaeM, BaeN, and BaeR contain more than 10 malonylation sites. BaeR is the most highly malonylated protein carrying 17 malonylation sites (Fan et al., 2017b,c).

Together with the data obtained for acetylation, the high malonylation rate of PKSs and NRPSs indicates a potential effect of protein modification on biosynthesis of antibiotics in FZB42. Better understanding of the underlying mechanism of how PTM affects PKSs and NRPSs may facilitate the development of FZB42 antibiotic production and application.

## AmyloWiki, an Integrating Data Base for FZB42

**FIGURE 4:**
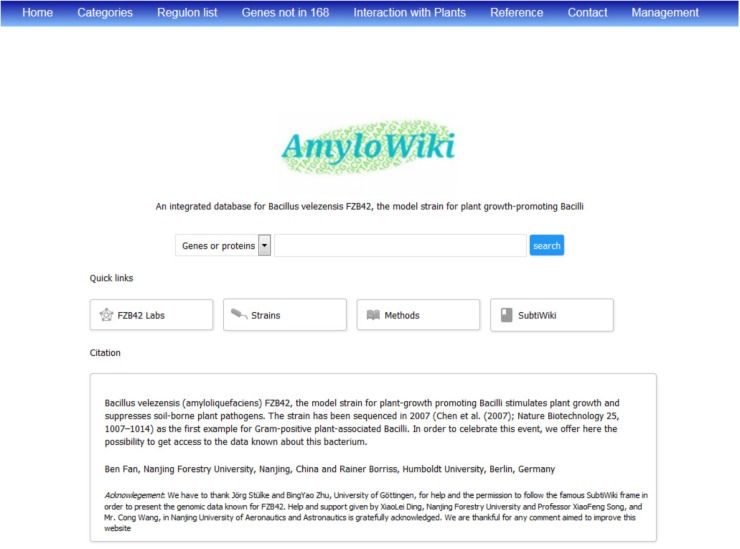
Home page of AmyloWiki, an integrated data base of FZB42.

With the increasing reception of FZB42 as a model organism for Gram-positive PGPR, and in order to celebrate its whole genome sequencing around 10 years ago (Chen et al., 2007), we have established an integrated database ‘AmyloWiki’^4^ for collecting and gathering all the information known to date about this bacterium (**Figure 4**). More than 140 articles about FZB42 can be found in AmyloWiki^5^ and are in part assigned to the corresponding genes/proteins. AmyloWiki centers the achievement of FZB42 studies till now including diverse information such as its 3979 genes, its transcriptome structure, protein regulators and their targets. 595 genes of FZB42 involved in plant-bacteria interactions were listed^6^. It informs also about recently identified sRNA genes and post-translational modification sites (see previous sections). A growing list of FZB42-site directed mutant strains, available for scientific community, is also presented. AmyloWiki shares some features with SubtiWiki, the popular database for *B. subtilis* 168 (Zhu and Stülke, 2018); however, specific features of FZB42 such as genes not occurring *in B. subtilis* 168, and genes involved in antagonism against plant pathogens and plant-microbe interaction, are highlighted in AmyloWiki. To facilitate communication and information exchange, a growing list of groups studying FZB42 is available, and many possibilities for interactive data exchange and feedback with the users are given.

AmyloWiki is configured to be a comprehensive and user-friendly database, built upon typical XAMPP (X-Windows, Linux or Mac OS + Apache + MySQL + PHP + Perl) environment. Apache 2.4.23 was used to construct a webserver. All data sets were processed and stored in MySQL (5.0.11). PHP language (version 5.6.28) was used to built database management system and interface. Webpages were designed with HTML5, CSS3 and JavaScript techniques. AmyloWiki provides a series of functions such as data submission, resource downloading, searching, advanced retrieval, and feedback.

Briefly, most information of user’s interest can be returned by performing a searching. User can search with different of the query strings, such as gene name, gene locus, and PubMed ID. The items that matched the query string will be returned in the result page. This can be exemplified by searching a gene, as happens most often. The basic information of the gene such as its product, locus, synonyms, homolog in *B. subtilis*, position, length and others, will be provided on the top of the result page. The genomic context of the gene can be viewed in a visualized window with scrollable function to check its neighbor genes. The organization of the gene, if it is present in an operon, the functions the gene involved, and its functional categories/subcategories are offered next. Other associated information includes the phenotypes of the mutant, its transcriptional start sites, protein/non-coding RNA regulators, sigma factors, PTM sites and so on. The references concerning the gene are listed at the bottom of the retrieval page.

For the convenience of the user, all datasets of AmyloWiki can be downloaded at the “Download” page. The data can be downloaded in an Excel-compatible format for their specific analysis. AmyloWiki will be maintained by us with a frequent update to improve its configuration and to keep the information comprehensive. For example, it is planned to add in future experimental protocols specifically worked out and used for FZB42, like transformation and bioassay. Here, support given by experienced groups dealing with FZB42 is highly welcomed. The pages for data submission and correction are designed for authorized users in order to update relevant information. Unauthorized users are encouraged to submit their latest data *via* E-mail to the authors of the website. Then their information will be verified and included in AmyloWiki.

## Conclusion and Outlook

In order to improve consistency in performance of bioinoculants in a sustainable agriculture we have to integrate them as part of modern crop management programs allowing to decrease the amount of agrochemicals, including harmful chemopesticides. A full understanding of the complex relationship between plant, soil, climate, microbiota, and the microbial inoculant is a necessary precondition for application success of biologicals. Basic research which has been restricted in past to selected representatives of taxonomic groups (‘model organisms’) such as *B. subtilis* 168, *E. coli* K12, *Saccharomyces cerevisiae*, and *Drosophila melanogaster* has considerably deepen our understanding of those groups in general. We recommend using FZB42 as a model for research on Gram-positive rhizobacteria. This will greatly enhance scientific progress in the field and might contribute to a better consistency in application of environmental friendly beneficial Bacilli in modern agriculture. After 20 years of basic and applied research FZB42 has been proven as suitable for selecting to this task. The following features favor use of FZB42 as model organism:

(1) Apathogenicity: Concerning biosafety issues, no representatives of the *B. subtilis* species complex including *B. velezensi*s have been listed as risk group in ‘The Approved List of biological agents’ (Advisory Committee on Dangerous Pathogens, 2013). However, *B. cereus* and *B. anthracis* were listed in human pathogen hazard group 3, excluding their use as biocontrol agents in agriculture.
(2) long term successful application of a commercialized product based on FZB42 in agriculture
(3) genetic amenable and a large collection of defined mutant is available for interested scientists
(4) large body of scientific knowledge (>140 articles) about FZB42 is already available
(5) an integrative data base ‘AmyloWiki’ has been established about FZB42 aimed to enhance collaboration of several groups dealing with Gram-positive PGPR and biocontrol bacteria

Most of the biocontrol agents currently in use are based on living microbes. Representatives of the *B. subtilis* species complex, including *B. velezensis*, *B. subtilis*, and *B. pumilus* are increasingly used for commercial production of biofungicides (Borriss, 2016). Most of them are stabilized liquid suspensions or dried formulations prepared from durable endo-spores. They are developed for seed coating, soil or leave application. Unfortunately, it is very unlikely that concentration of *Bacillus* synthesized cyclic lipopeptides in their natural environment is sufficient for antibiosis (Debois et al., 2014). A possibility for circumventing this problem are combined bioformulations consisting of both, *Bacillus* spores and antagonistic acting metabolites. However, only a few bioformulations currently on the market, such as SERENADE^®^ prepared from *B. subtilis* QST713 and Double Nickel 55 prepared from *B. amyloliquefaciens* D747 (both strain names need to be corrected as *B. velezensis*, Fan et al., 2017a), contain together with living spores antimicrobial compounds, such as cyclic lipopeptides (iturins, fengycin). Unfortunately, also in these products only the number of spores is declared as active ingredient of the biofungicide, but concentration of the metabolites is not indicated, excluding an exact treatment of pathogen infected plant parts. Labeling a fixed concentration of the active principle for suppressing the target would allow a better comparison of chemical and biological pesticides (Borriss, 2015b). To the best of our knowledge, no bioformulations containing exclusively antimicrobial metabolites are commercially available, although companies like ABiTEP performed extended large scale trials with concentrated and stabilized *Bacillus* supernatants in order to suppress plant pathogens.

## Author Contributions

BF and RB outlined and wrote the manuscript. BF, CW, XLD, XFS, and RB developed the integrated data base AmyloWiki. LW, HW, and XG contributed essential scientific results reported in this review. All authors have approved and corrected the final version of the manuscript.

## Conflict of Interest Statement

The authors declare that the research was conducted in the absence of any commercial or financial relationships that could be construed as a potential conflict of interest.

## Footnotes

1 http://amylowiki.top/reference.php

2 https://www.abitep.de/index.php/de/agrar.html

3 http://amylowiki.top/interaction.php

4 http://amylowiki.top/

5 http://amylowiki.top/reference.php

6 http://amylowiki.top/interaction.php
