# Corrigendum: *Bacillus velezensis* FZB42 in 2018: The Gram-Positive Model Strain for Plant Growth Promotion and Biocontrol

**DOI:** 10.3389/fmicb.2019.01279

**Published:** 2019-06-11

**Authors:** Ben Fan, Cong Wang, Xiaofeng Song, Xiaolei Ding, Liming Wu, Huijun Wu, Xuewen Gao, Rainer Borriss

**Affiliations:** ^1^Co-Innovation Center for Sustainable Forestry in Southern China, College of Forestry, Nanjing Forestry University, Nanjing, China; ^2^Department of Biomedical Engineering, Nanjing University of Aeronautics and Astronautics, Nanjing, China; ^3^Department of Plant Pathology, College of Plant Protection, Nanjing Agricultural University, and Key Laboratory of Integrated Management of Crop Diseases and Pests, Ministry of Education, Nanjing, China; ^4^Institut für Biologie, Humboldt Universität Berlin, Berlin, Germany; ^5^Nord Reet UG, Greifswald, Germany

**Keywords:** *Bacillus velezensis*, FZB42, AmyloWiki, induced systemic resistance (ISR), non-ribosomal synthesized lipopeptides (NRPS), non-ribosomal synthesized polyketides (PKS), volatiles, plant growth promoting bacteria (PGPR)

In the original article, there was an error in referring to the approved group of *Bacillus cereus*.

A correction has been made to the **Conclusion and Outlook** section, “(1) Apathogenicity”:

“(1) Apathogenicity: Concerning biosafety issues, no representatives of the *B. subtilis* species complex including *B. velezensi*s have been listed as risk group in ‘The Approved List of biological agents’ (Advisory Committee on Dangerous Pathogens, 2013). By contrast, use of strains of *B. cereus* needs special attention, since they are a member of risk group 2 and closely related to *B. anthracis*, a human pathogen and the member of risk group 3.”

The authors apologize for this error and state that this does not change the scientific conclusions of the article in any way.
